# Essentials of the Principals and Practice of Medicine

**Published:** 1875-01

**Authors:** 


### Books Reviewed.

*Essentials of the Principles and Practice of Medicine. A Hand-Book for Students and Practitioners.* By Henry Hartshorne, A. M. M. D. Fourth Edition. Thoroughly Revised. Philadelphia: Henry C. Lea, 1874. Buffalo: T. Butler & Son.

Dr. Hartshorne's little work has ever since the appearance of the first edition, been a favorite with students and a large number of practitioners. The present edition will be found materially changed from the last one. Some new topics have been introduced, and the addition of illustrations to the number of one hundred will serve better to elucidate the text. Among the topics which are for the first time introduced in this edition of the work are, cold baths in fever, transfusion of blood, and recto-abdominal exploration. A few remarks have also been introduced upon the diagnosis and treatment of such forms of the diseases of women as fall under the observation of the general practitioner. Of a book so well known by former editions it is unnecessary for us to speak at length, we need only say that it is highly worthy of the favor accorded to former issues.

